# Supplementary material for: Patterns of Intron Gain and Loss in Fungi
Source: PLoS Biol. 2004 Nov 30;2(12):e422. doi: 10.1371/journal.pbio.0020422 (PMC532390; doi:10.1371/journal.pbio.0020422)
Supplement: Table S1 — Also available at http://genes.mit.edu/NielsenEtAl/. (4.3 MB ZIP). [file pbio.0020422.st001.zip › NielsenEtAl/html/982.html]

AN7111.1.NCU08828.1.MG06148.1.FG09643.1


```
 CLUSTAL W (1.82) Multiple Sequence Alignments - Introns Inserted


Sequence 1: NCU08828.1	894 aa
Sequence 2: MG06148.1	896 aa
Sequence 3: FG09643.1	899 aa
Sequence 4: AN7111.1	883 aa
Alignment Length: 906 aa
Number Identitical Residues: 530 aa
Alignment Score (without introns) 25056


MG06148.1 	MAAELRFDGQVVVVTGAGGGLGKAYATFFGSRGASVVVNDLGGSFKGEGTSSK0AADVVV
NCU08828.1	MAEQLRFDGQVVVVTGAGGGLGKAYCLFFGSRGASVVVNDLGASFKGEGNSTK0AADVVV
FG09643.1 	MAEQLRYDGQVVVVTGAGGGLGKAYATFFGSRGASVVVNDLGVTSKGEGNSSK0AADVVV
AN7111.1  	-MSELRFDNQTVVVTGAGGGLGKAYALFFASRGANVVVNDLGGSHQGEGKSSK0AADVVV
          	   :**:*.*.**************. **.****.******* : :***.*:* ******

MG06148.1 	NEIKAAGGKAVANYDSVENGDKIIDTAIQAFGRIDILINNAGILRD~ISFKNMSDQDWDL
NCU08828.1	NEIKAAGGKAVANYDSVENGDKIIETAIKEFGRIDILINNAGILRD~ISFKNMKDEDWDL
FG09643.1 	NEIKAAGGKAVANYDSVENGERIIETAISAFGRIDILINNAGILRD~ISFKNMKDEDWDL
AN7111.1  	DEIRAAGGKAVANYDSVENGDAIIDTAIKNFGRVDVLINNAGILRD0EPVLRRSTDER-L
          	:**:****************: **:***. ***:*:**********  .. . . ::  *

MG06148.1 	IFKVHVKGAYKCARAAWPHFRKQKYGRVINTASAAGLFGNFGQCNYSA~AKLAMVGFTET
NCU08828.1	IFKVHVKGSYKTARAAWPYFRKQKFGRVINTASAAGLFGNFGQANYSA~AKLGMVGFTET
FG09643.1 	IYKVHIKGSYKCARAAWPHFRKQKYGRVINTASAAGLFGNFGQANYSA~AKLAMVGFTET
AN7111.1  	IS--------QCARAAWPHFRKQKYGRIINTASAAGLFGNFGQANYAA1AKLGQVGFTET
          	*         : ******:*****:**:***************.**:* ***. ******

MG06148.1 	LAKEGVKYNILANVIAPIA~ASRMTETVMPPDLLALMKPEWVVPLVAVLVHKNNTNETGG
NCU08828.1	LAKEGLKYNIISNVIAPIA~ASRMTETVMPPDLLALMKPEWVVPLVAVLVHKNNTSETGS
FG09643.1 	LAKEGIKYGILANVIAPVA1ASRMTETIMPPDVLEQLKPEWVVPLVAVLVHKNNTQETGG
AN7111.1  	LAKEGAKYNIIANVIAPIA1ASRMTATVMPPEVLELLKPEWVVPVVAVLVHSSNTTESGS
          	***** **.*::*****:* ***** *:***::*  :*******:******..** *:*.

MG06148.1 	IFEVGGGHCAKLRWERSSGLLLKCDETYTPGAIIKKWNQVVDFSKPQYPSGPNDFLSLLE
NCU08828.1	IFEVGGGHVAKLRWERSSGLLLKADESYTPGAIIKKWDQVTDFSNPQYPTGPNDFLALLE
FG09643.1 	IFEVGGGHVAKLRWERSSGLLLKADDSYTPGAIIKNWGKVVDYSNPQYPTGPNDFLTLLE
AN7111.1  	IFEIGGGHVAKLRWERSKGALLKTDASLTPGAIARKWNEVNDFSKPDYPTGPADFMGLLE
          	***:**** ********.* *** * : ***** ::*.:* *:*:*:**:** **: ***

MG06148.1 	ESTQLGPNDKGEPIDYKGRVALVTGGGAG~IGRAYCLAFARGGASVVVNDLANPDGVVNE
NCU08828.1	ESLKLGPNDPGEKVDFKGRVALVTGGGAG~IGRAYCLAFARAGASVVVNDLVNPDDVVNE
FG09643.1 	DSMKMGPAEQGENPDFTGRVALVTGGGAG~IGRIYALAFAKYGASVVVNDLADPEPVVAE
AN7111.1  	DGLKLPPAPSGPEPEFKGKVALVTGGGNG2LG-----------AKVVVNDLVDPEPVVQE
          	:. :: *   *   ::.*:******** * :*           *.******.:*: ** *

MG06148.1 	IKQMGGKAIGIKASAEDGDAVVKAAIDAFGRIDIIINNAGILRDKAFTNMDDNLWDPVMN
NCU08828.1	IKKMGGKAVGAKFSAEDGDAVVKAAIDAFGRVDIVVNNAGILRDKAFHNMDDSLWDPVMN
FG09643.1 	IKKLGGKAVGVKASAEDGEKVVKAAIDAFGRIDIVVNNAGILRDKAFSNMNDDLWDPVLN
AN7111.1  	IKKAGGEAVGNKASCEDGAAVVKTAIDTYGRIDILVNNAGILRDKAFTNMNDDLWNPVVN
          	**: **:*:* * *.***  ***:***::**:**::*********** **:*.**:**:*

MG06148.1 	VHARGTYKITKAAWPYLLKQKYGRIVNTTSTSGIYGNFGQANYSAA0KCAILGFSRALAL
NCU08828.1	VHARGTYKVTKAAWPYFLKQKYGRVLNTTSTSGIYGNFGQANYSAA0KCAILGFSRAIAL
FG09643.1 	VHLRGTYKVTKAAWPYFLKQKYGRVLNTTSTSGIYGNFGQANYAAA0KCGILGFSRALAL
AN7111.1  	IHLRGTYKVTQAAWPHMLKQKYGRIVNTASTSGIYGNFGQANYAAA0KLGILGFSRALAI
          	:* *****:*:****::*******::**:**************:** * .*******:*:

MG06148.1 	EGAKYNIFVNTIAPNAGTAMTKTILPEELVQAFKPEYIAPLVLALCSDKVPE-NPTGGLY
NCU08828.1	EGAKYNIYVNTIAPNAGTAMTKTILPEELVQAFKPDYVAPLVLALCSDKVPK-KPTGGLY
FG09643.1 	EGHKYGIYVNTIAPNAGTAMTATIMPDEMVQAFKPDYIAPLVLALCSDKCP--NPTGGLY
AN7111.1  	EGAKYNIKVNTIAPNAGTNMTRTIMPEEMVQAFKPDYVAPLVVLLCSDMTPEPYSTKGLF
          	** **.* ********** ** **:*:*:******:*:****: ****  *.. .* **:

MG06148.1 	EVGSGWQGATRWQRSGGHGFPVDVPLTPEEVAKNWARIVNFDDGRADHPFKSQDSVAKIM
NCU08828.1	EVGSGWCGQTRWQRSGGHGFPVDVPLTPEEVVKHWNDIVTFDS-RADHPEKASDSIEKIM
FG09643.1 	EVGSGWCGQTRWQRTGGHGFPVDVALVPEEVVKHWKDIVTFDG-RADHPVKSQDSIEKIM
AN7111.1  	ECGSGWFGSTRWQRSGGHGFPVDVKLTPEAVAKELAKIVNFDDGRADHPEDIQAANEKVM
          	* **** * *****:********* *.** *.*.   **.**..***** . . :  *:*

MG06148.1 	ENMDNKAGSK0SG~SVSGSNQYLEAIQRAKDAKTEGTEFVYSEKDSILYNLGIGAKRTDL
NCU08828.1	ANMENRVG-E~GK0SGAAENEHLAAIKKFTGVEGKGTEYTFTERDVCLYNLGIGAKRTDI
FG09643.1 	ANMQNTSK--~GK~ESSGETDYKKVIAETVTKEGKPTEFKYEERDVILYNLGVGAKRTDL
AN7111.1  	SNFSNRSG--~GE~SGGSGNEILSAIEAAKKASTDGTSFDYTDRDVILYNLSLGAKRTDL
          	 *:.*      .  . .. .:   .*      . . *.: : ::*  ****.:******:

MG06148.1 	DYVY2EGAEDFQVLPTFGVIPQFNADMPFSMDEVVPNFNPMMLLHGEQYLEIKKYPIPTS
NCU08828.1	KYIF~EGNEDFEVVPTFGVIPPFNTEMPFSFDDIVPNFSPMMLLHGEQYLEVRKYPIPTS
FG09643.1 	KYVF~EGSEDFQVLPTFGVIPPFNTEMPFDFDNIVPNFSPMMLLHGEQYLEIRKFPIPTN
AN7111.1  	PLVY~ENNEHFQALPTFAVIPWFNTATPWDMNDIVKNFSPMMLLHGEQYMEIRKFPIPTE
          	  :: *. *.*:.:***.*** **:  *:.::::* **.**********:*::*:****.

MG06148.1 	AKLKSYGKLLEVVDKGNAAVLRNGVTTVHAETGEEIFYNEASIFLRGSGGFGG-PKKAQD
NCU08828.1	GRLVSKGKLLEVVDKGSAAIVKQGITTFNAETGEELFYNEMTVFLRGCGGFGG-QKKPAD
FG09643.1 	ARLVSRGRLLEVVDKGNASIARSSTTTVDANTGEDVFYNEASVFLRGTGGWGG-PKRGAD
AN7111.1  	AKTKTYPKLIDVIDKGAAALVVAGYTTKDVATGEDLFYNESTVFIRGSGGFGGSPKPTAP
          	.:  :  :*::*:*** *::   . ** .. ***::**** ::*:** **:**: *    

MG06148.1 	R-GASTAANTPPKRSPDVVVEEKTTEEQACIYRLSGDYNPLHVDPQFAKMGGFPQPILHG
NCU08828.1	R-GASTAANKPPARSPDAVVEVQTTEEQAAIYRLSGDYNPLHVDPAFAKVGGFKVPILHG
FG09643.1 	R-GSATAANKPPARAPDVVVESPTNDDQAAIYRLSGDYNPLHIDPEFAKVGGFKAPILHG
AN7111.1  	RPKAAVAAYKPPKRKADVVVEEKTSEDQAALYRLNGDRNPLHIDPEFSKVGGFKIPILHG
          	*. ::.** .** * .*.***  *.::**.:***.** ****:** *:*:***  *****

MG06148.1 	LCFFGISGKAVYQQFGKIKNIKVRFAGVVMPGQTLVTEMWKEGNKIIFQTKVKETGKLAI
NCU08828.1	LCSFGIAGKAVYEKYGKFKNIKVRFAGTVNPGQTLVTEMWKEGNKVVFQTKVKETGKLAI
FG09643.1 	LCSFGVAGKAVYERFGAFKNIKVRFAGVVIPGQTIVTEMWREGNKIIFQSKVKETGKPAI
AN7111.1  	LCSLGVSGKHVFQKFGPIKNLKVRFAGVVLPGQTLRTEMWKEGNTVLFQTTVVETGKPAI
          	** :*::** *::::* :**:******.* ****: ****:***.::**:.* **** **

MG06148.1 	GGAAAELY-------
NCU08828.1	SGAAAELA-------
FG09643.1 	AGAAAELRTDGKSKL
AN7111.1  	SGAGAELLEGAKAKL
          	.**.***  ...:.
```
